# Understanding the mental health and recovery needs of Canadian youth with mental health disorders: a Strategy for Patient-Oriented Research (SPOR) collaboration protocol

**DOI:** 10.1186/s13033-019-0264-0

**Published:** 2019-01-31

**Authors:** Skye P. Barbic, Adelena Leon, Ian Manion, Sarah Irving, Rebecca Zivanovic, Emily Jenkins, Shelly Ben-David, Pouya Azar, Amy Salmon, Carolyn Helps, Stephanie Gillingham, Tara Beaulieu, Rachal Pattison, Corinne Talon, Oluseyi Oyedele, Karen Tee, Steve Mathias

**Affiliations:** 1 Faculty of Medicine, University of British Columbia, Vancouver, BC Canada; 2 Department of Occupational Science and Occupational Therapy, Faculty of Medicine, The University of British Columbia UBC, Vancouver Campus, TF297-2211 Wesbrook Mall, Vancouver, BC V6T 2A1 Canada; 3 Department of Psychiatry, UBC, Vancouver, BC Canada; 4 Centre for Health Evaluation and Outcome Sciences, Vancouver, BC Canada; 5 Foundry Research and Innovation, Vancouver, BC Canada; 6 University of Ottawa, Ottawa, Canada; 7 FRAYME, Ottawa, Canada; 8 Canadian Mental Health Association, Vancouver, BC Canada; 9 School of Nursing, UBC, Vancouver, BC Canada; 10 Department of Psychiatry, Providence Health Care, Vancouver, BC Canada; 11 University of Victoria, Victoria, BC Canada; 12 British Columbia Centre on Substance Use, Vancouver, BC Canada

**Keywords:** Young adult, Mental health, Measurement, Patient engagement

## Abstract

**Background:**

While considerable progress is being made to understand the health and self-management needs of youth with mental health disorders, little attention has focused on the mental health and recovery needs that the youth themselves identify—this despite a national priority to incorporate patient-oriented research into the development and assessment of mental health services. To address this gap, estimates of the extent to which existing patient-reported outcome measures (PROMs)—originally developed for use amongst adult populations—are clinically meaningful and psychometrically fit for use among youth are needed. In tandem, a recovery profile for youth can be constructed incorporating the youth perspective of the services provided within a community mental health setting.

**Methods/design:**

This study will utilize a mixed methods design incorporating qualitative focus group interviews and cross-sectional survey. Our process will begin with the hiring of a youth peer research partner who will provide lived experience expertise through all phases of the study. We will advertise, recruit, and conduct four focus groups with youth who receive services from the Foundry Vancouver Granville located in British Columbia, Canada. In the first two focus groups, we will recruit youth aged 15–18 years (n = 10). In the second two focus groups, we will recruit young adults aged 19–24 years (n = 10). In parallel, we will conduct a cross-sectional survey of the recovery and mental health needs of youth, informed by ten widely used and validated PROM. Thematic analysis techniques will guide the identification of predominant thematic trends in the qualitative focus group data. We will use Classical and Rasch measurement methods to test and analyze the reliability and validity of selected PROM measures for youth populations.

**Discussion:**

The proposed study has the potential to produce a preliminary conceptual and measurement model for understanding the mental health and recovery needs of youth with mental health disorders. This evidence will inform how youth mental health services can grow, support, and sustain the capacity for a collaborative, interdisciplinary and innovative patient-oriented research environment. Findings will also contribute much needed evidence to improve the standard of care for youth who experience mental health disorders in Canada and beyond.

## Introduction

Mental health disorders affect approximately one in four Canadian youth [1, 2], with those aged 12–24 experiencing the highest incidence of mental disorders among any age group [3, 4]. Adolescence and early adulthood are considered the peak periods for the onset of mental disorders, with 75% of all diagnoses having an onset before age 25 [5, 6]. With data estimating the economic burden of mental health disorders in Canada at $51 billion annually [7, 8], there is an urgent need to re-examine services for this high priority group [8–12]. Paradoxically, it is during adolescence and young adulthood that access to mental health services is the poorest [13] and current mental health services are “largely inadequate and unsuited to [youth] needs” [12, 14]. To reduce the impact of mental health disorders on Canadian youth, transformative change and service redesign are necessary.

In September 2014, we submitted a proposal entitled *Transforming Access to Health and Social Services for Transition*-*Aged Youth (12*–*25)* to the Select Standing Committee on Children and Youth in British Columbia (BC), Canada. The proposal called for the creation of a network of health and social service centres across BC that would provide youth- and family-centred services to youth with mental health disorders. This proposal resulted in the establishment of Foundry and the first six Foundry centres, which provide integrated mental health and substance use services, primary care, social services and youth and family peer supports to youth aged 12–24 (foundrybc.ca). With the goal of implementing timely, evidence-based, and youth-centred services across the province, our team recognized early that there is a significant gap in evidence on the recovery and mental health needs of youth, identified by youth themselves [15, 16]. We also identified a lack of measurement tools fit to measure recovery and mental health outcomes for youth.

In Canada, the term “recovery” in mental health care extends beyond symptom reduction to emphasize outcomes associated with *“a way of living a satisfying, hopeful, and contributing life, even with the limitations caused by illness*” [17]. This dates back to the late 1980’s, when the patient-led recovery movement was established [17, 18], with the purpose of providing a voice for patients with mental health disorders, and emphasised health rather than illness. Recovery and “health” have become overarching goals for mental health service provision globally, and in the last decade they have become the primary objectives of mental health system reforms in Canada. Service organizations now face increasing pressure to articulate how their interventions and outcomes fit within a person-centred, recovery-oriented framework [19–21]. Although the principles of person-centred care are gaining acceptance for adults with mental health disorders in Canada, less attention is centred on the unique needs and priorities of youth. For instance, despite the emphasis on providing mental health services that align with person-centred principles, a recent systematic review identified that youth are “rarely actively involved” in their mental health treatment [22]. Moreover, consensus on the definitions of terms such as “health”, “mental health”, and “recovery” remains illusive [23]. To advance the science of patient-centred youth mental health care, stakeholders in the field must clearly identify, conceptualize, and prioritize targeted outcomes of care. Clear targets will be important to help stakeholders learn to systematically measure outcomes and to drive services according to the needs of youth.

One challenge with measuring youth outcomes in mental health care is that they are often not directly observable—they have to do with how youth feel, function, and perceive wellness and health. To infer the extent to which treatments and health services are effective, data need to be gathered from patients [24]. Often this data is gathered utilizing patient-reported outcome measures (PROMs). PROMs have the potential to capture outcomes such as sustained symptom reduction, return to functioning, and optimization of patient mental health and recovery [21, 25]. Unfortunately, as the demand increases for accountability of a broad range of mental health services to be patient-centered, there is limited consensus on rating scales fit to measure these outcomes for youth [26, 27]. Successful youth engagement in the development of conceptually-driven measurement models are needed to ensure that research and clinical innovation in youth mental health services in Canada are relevant for this patient population.

The overall goal of this project is to address a national priority to incorporate patient-oriented research into the development and assessment of community mental health services for youth. The specific research aims are to:

1. Understand the mental health and recovery needs of Canadian youth, *as identified by youth*;
2. Use existing outcome assessments designed to measure mental health and recovery in adults to determine what evidence can be gleaned about the recovery profile of youth;
3. Estimate the extent to which available PROMs are clinically meaningful and psychometrically fit for purpose to measure the mental health and recovery needs of youth.

## Methods

To meet the objectives of this project, we will use Canada’s Strategy for Patient-Oriented Research (SPOR) Patient Engagement Framework [28] and the recent Emerging Guidelines for Patient Engagement in Research [29, 30] throughout the four phases of this mixed methods study. The total duration of the study is 1 year. We will also use the core values of youth- centred care at Foundry to guide study design and procedures. We will obtain ethical approval at relevant institutions for all parts of this study.

### Phase 1. Hiring and Training a Youth Patient Research Partner (PRP) and Youth Research Assistant (YRA) (months 1–2)

Patient and family engagement strategies have been formally embedded in Foundry’s organizational structure since its initial development stages, including at its primary care site, Foundry Vancouver Granville centre. During this first phase, our team will develop a job description in consultation with knowledge users to recruit a youth Patient Research Partner (PRP) and Youth Research Assistant (YRA). As recommended by Kirwan et al. [31], we will seek to identify candidates with strong communication skills, motivation, and assertiveness in a team setting. The research lead will ensure that YRA will be recognized in the project, including publications and knowledge translation activities.

Upon hiring of the PRP and YRA, the knowledge users (researchers, Foundry leadership) will create a safe environment that promotes honest interactions, cultural competence, training, and education. Support will also involve financial compensation for the PRP’s involvement, with the PRP hired officially as a staff member on the study team. We will document shared learnings about the strengths and limitations of the hiring process and report any concerns to leadership. Once the entire team is established, we will hold a team meeting to outline project details, develop and sign a team contract outlining members’ roles, and co-create a vision of team values, including the key principle of mutual respect for all team members and a commitment to collaboration and excellence.

### Phase 2. Identification of recovery needs of youth, from the perspective of youth and young adults (months 4–6)

We will advertise and recruit four focus groups, which we will conduct with youth participants who receive services from Foundry Vancouver Granville. In focus groups 1 and 2, youth aged 15–18 years will be recruited (n = 10), while groups 3 and 4 will have young adults aged 19–24 years (n = 10). If youth are under the age of 19 years and assent is required, the PRP will meet with the youth and their parent or designated caregiver. If no parent or caregiver is available to consent for the youth, we will consider ethical issues of assent guided by the Behavioral Research Ethics Board at the University of British Columbia. This will include having youth review an assent form written in lay terms, that clearly explains what the study is about and why it is being done, and spells out and explains any acronyms in simple terms. As well, the assent form will also explain summarily and accurately what the participant will have to go through, including the total time that the participants will spend participating in the study and who is doing the study, who to contact if they have questions, and information about confidentiality.

Focus group questions will include: (1) *What does health and recovery mean to you?*; (2) *What does it look like to go from low to high recovery?*; (3) *What supports are needed to help you achieve your health and recovery goals in the short*-*term?*; and (4) *What supports are needed to help you achieve your health and recovery goals long*-*term?.* The PRP and YRA for the study will moderate the focus groups with the assistance of the research lead. These group interviews will be audio-recorded, transcribed without identifiers and uploaded to NVivo 10 qualitative management software to facilitate analysis. Inductive thematic analysis [23] will identify themes within participants’ experiences with mental health services and recovery, along with their perceived needs to achieve short- and long-term recovery. Two members of the research team with expertise in qualitative analysis will independently review and generate a series of broad codes. The study team will then collectively organize codes into themes to be compared and contrasted within and across focus groups. Initial themes derived from the data will be verified by the multidisciplinary research team and study participants, using an iterative process to refine participant perspectives on what is required to support their recovery needs. Comprehensive reporting of all analysis stages will be framed using the Consolidated Criteria for Reporting Qualitative Research (COREQ) [32].

### Phase 3. PROMs: assessment of the recovery and mental health needs of youth (months 6–10)

#### Recruitment

Recruitment will occur at Foundry Vancouver Granville centre, which receives 1200 visits per year from youth aged 12–24. Staff at the centre will support recruitment of participants in two ways: (1) they will post flyers about the project in high traffic areas in the centre; and (2) they will schedule times for a research team member to be present in the clinic to meet potential participants and provide study information.

#### Procedures

Interested participants, aged 15–24 years, who speak English, will meet with the PRP who will answer questions about the study. We will obtain informed consent prior to the study. After consent is obtained, we will give the participant a study assessment package. As noted above, if youth are under the age of 19 years and assent is required, the PRP will meet with the youth and their parent or designated caregiver or follow guidelines for assent. The study assessment package will include two paper copies of the consent form, a study participant number, and a tablet with the demographic questionnaire and PROMs programmed in (See Table 1 for list of patient-reported outcomes included). Pilot testing of the included survey has been completed by a pilot group of ten youth who receive mental health services from Foundry. Overall, youth in the pilot testing enjoyed this form of administration over the paper version and felt comfortable with the data collection platform [33]. Youth also enjoyed with engaging with a YRA. As a result, the YRA will be responsible for all procedures related to recruitment, consenting, and data collection. Moving forward, data will be collected via a secure web-based application, Research Electronic Data Capture (REDCap), hosted by the Canadian HIV Trials Network. Participants will be compensated $15 (Canadian) for their time, which is estimated at 30 min.

**Table 1:** Summary of the rating scales used for the proposed study

Scale	Abbr	Description
Mental health		
Kessler Psychological Distress Scale [34]	K10	This is a 10-item questionnaire intended to yield a global measure of distress based on questions about anxiety and depressive symptoms that a person has experienced in the most recent 4-week period [35, 36]
Patient Health Questionnaire [37]	PHQ-9	This screening tool is commonly used to screen for depression. The PHQ-9 has been shown to have 61% sensitivity and 94% specificity in adults [38–40]
General Anxiety Disorder Scale [41–43]	GAD-7	A self-report questionnaire with 7 items for screening and severity measuring of generalized anxiety disorder. Assessment is indicated by a total score, which is made up by adding together the scores for the scale of all seven items. The tools has been validated primarily in adults [41, 43, 44]
Beck Anxiety Inventory [45]	BAI	The BAI is a 21-item multiple-choice self-report inventory that measures the severity of an anxiety in adults and adolescents. The BAI items emotional, physiological, and cognitive symptoms of anxiety but not depression. The BAI has been used with young adults and can be completed in 5 min. The BAI has been shown to be psychometrically strong with high internal consistency (Cronback alphas ranging from 0.92 to 0.94) and test–retest reliability is 0.75. With youth, the BAI has also been shown to possess acceptable reliability and convergent and discriminant validity for both 14–18 year and inpatients and outpatients [46, 47]
Global Appraisal of Individual Needs– Short Screener [48]	GAIN-SS	The GAIN-SS is a short screening tool used in general populations to quickly identify people who would be flagged as having a behavioral health disorder. The measure has four subscales, each shown to work as a unidimensional measure [49]. We are unaware of any studies to date that have tested the GAIN-SS explicitly with young adults receiving community mental health services
Recovery		
Canadian Personal Recovery Outcome Measure (PROM) [50]	C-PROM	New 30-item self-report scale to measure recovery in Canadians with mental illness. Measure has been shown to have high internal consistency, excellent face validity, and adequate test–retest repeatability with adults [51]. Has been tested with 200 young adults aged 18–30 years showing preliminary evidence to be fit for purpose for this population group. This study will provide further evidence to support the PROM’s utility and psychometric validity with youth
The Illness Management and Recovery Scale [52]	IMR	The IMR is a 13-item self-report measure of self-management and pursuit of recovery goals. Each item has a unique response set. The IMR has adequate internal reliability (α = 0.72) and good test-re-test reliability (α = 0.81) [52]
Quality of Life		
EQ-5D [53]	EQ-5D	The EQ-5D measures health related quality of life (HRQL). It provides a utility between 0 and 1 representing the value placed on life lived in the current health state and is optimized for economic analyses and comparisons across health conditions. A youth version of the EQ-5D has been developed [54] and validated internationally [55]
Recovery Quality of Life Questionnaire-10 item version [56, 57]	ReQOL-10	The ReQOL is a newly developed measure of ReQoL-10 contains positively and negatively worded items covering seven themes: activity, hope, belonging and relationships, self-perception, well-being, autonomy, and physical health. The measure has been shown to have acceptable internal consistency, test–retest reliability (Cronbach alphas > 0.85), known-group differences, convergence with related measures, and were responsive over time (standardised response mean (SRM) > 0.4) [57]. The measure has not yet been validated with young adults receiving community mental health services

### Phase 4. Psychometric testing of PROMs (months 10–12)

The aim of this phase is to pilot test and estimate the extent to which the existing PROMs are fit for purpose to measure recovery and mental health of the youth with mental health disorders receiving Foundry services. A set of ten PROMs have been identified with global and regional expert consultation. The PROMs to be tested are listed in Table 1. The results from this section will inform the selection of measures for a larger scale multi-centre psychometric study in 2019. We will use Classical and Rasch measurement theory approaches to assess the psychometric properties each PROM for score reliability, construct validity, and ability to detect change.

#### Classical psychometric analysis

Data from the PROMs collected in Phase 3 will be examined for completeness, equality of variances, normality, scale to sample targeting (score means; standard deviations [SD]; floor and ceiling effects), internal consistency (Cronbach’s alpha), and magnitude of item-total correlations [58]. Sociodemographic characteristics of participants will be compared by employing independent-sample t-tests and Chi square goodness-of-fit tests. Convergence and discriminant construct validity will be tested by examining correlations between the PROMs tested and demographic variables (gender, age, ethnicity and diagnosis).

##### Rasch measurement testing

Responses will also be analyzed using modern psychometric methods. A confirmatory analysis will be carried out, followed by Rasch analysis [59, 60] which tests the extent to which the items from each PROM form a linear continuum. The ordering of the response options will be verified, and the unique location of each item estimated to identify redundant items. We will use RUMM 2030 as the software for the analysis [61]. A minimum sample size of 250 will provide stable person and item estimates of location on the continuum [62]. This sample size has been shown to be consistent across health fields [63–65], including recent research conducted by the research lead [24, 66, 67]. To be conservative we have set our target sample to 350.

## Discussion

Ours is the first study that we know of to explore and measure the health and recovery needs of youth with mental health disorders. Evidence generated from this study will build capacity for patient-oriented research in the area of youth mental health. Specifically, it will inform how youth mental health services can grow, support, and sustain a collaborative and interdisciplinary patient-oriented research environment. We will ensure the PRP is well integrated into the research process in the following capacities: longitudinal data collection, PROM development and testing, interpreting datasets, and encouraging networking and relationship building opportunities in the mental health research community. By including PRPs in all phases of this research, we will gain valuable knowledge about the impact, benefit, and feasibility of our approach for enhancing the quality of clinical care and service delivery for youth. This project will provide a preliminary model for Foundry and other Canadian youth services, guiding the implementation of collaborative research projects to shape clinical initiatives and future research based on youth-identified needs.

Researchers investigating health services in Canada are increasingly required to involve patients and their families at the core of their work. Our study team has included youth and family collaborators in the preparation of the research project (agenda formulation, grant writing, funding procurement), and there is commitment to maintaining this standard throughout the execution of this research (study conduct, data analysis, interpretation of results) and the translation of results into action. To do so, ongoing training will be provided to the PRP, YRA, and knowledge users, to ensure they are well equipped with the skills for meaningful engagement in patient-oriented research. Through weekly meetings, the aim will be to achieve excellence in training for all team members, thereby ensuring that the methodology and outcomes of this project produces high quality, rigorous research that brings real benefits for patients in their daily lives.

With the inception of Foundry, we see an unprecedented opportunity to work closely with youth research partners to design and evaluate innovative services that are tailored to meet the specific needs of youth living with mental health and substance use disorders in BC and beyond. By closely documenting our process and sharing our results in a timely manner, this preliminary work will catalyse new patient-oriented initiatives within Foundry. This collaborative approach will enhance the results of the proposed project and will lead to better study results that are realistic and patient\-centered. Most importantly, the results of this collaboration between youth, knowledge users, and researchers will contribute to improved quality of care for youth with mental health disorders living in Canada and beyond.

The anticipated outcomes of this project include

I. Developing a profile of the mental health and recovery needs of youth from the perspectives of patients.
  This profile of 350 Foundry youth (survey) and 40 youth (focus groups) will provide a comprehensive overview of the areas that youth consider to be important and priorities for mental health and recovery. Altogether, these results will guide Foundry and other Canadian youth mental health services to better understand and assist patients with their mental health and recovery goals. Also, this study will contribute evidence towards the feasibility of using PROMs in a clinical setting to identify and measure outcomes important to youth.
II. Create a culture for patient-oriented research for youth receiving mental health services in BC and Canada.
  As noted in Canada’s SPOR document (2015), “*health services research studies document a long history of advocating for more involvement of patients in decision research priorities and agendas, and there is increasing evidence on how valuable this can be”* (p. 16). Little documented evidence has been identified to support this statement among youth with mental health disorders. By creating a culture of youth engagement in this project, we will be able to guide how services are delivered and meet the needs of key stakeholders.
III. Develop preliminary evidence for a set of items that are fit for purpose to measure the recovery and mental health needs of youth.
  This study will provide preliminary evidence for understanding how current measures of mental health and recovery can collectively be used as reliable and valid instruments for youth. The psychometric summary of the proposed measures will be a starting point to understand the conceptual underpinnings and measurement of mental health and personal recovery in youth. This information will be used to design future research studies that will develop and test outcome assessments that are fit for purpose for youth. Alternatively, should the outcome assessments be shown to be reliable and valid, this evidence can then be used by key stakeholders and researchers within Foundry to understand the recovery trajectories of youth who receive Foundry services. In this study, we will also gain valuable information from our Information Technology collaborators to implement relevant measures into the current and future clinical data collection systems at Foundry to enhance clinical practice and patient-centred care.
IV. Bridge the research-to-practice valley through the establishment of a Research Steering Committee and establishment of a culture for patient-oriented research.
  In October 2016, Foundry started a Research Steering Committee to guide research priorities and ensure the methodological rigor of research stemming from the initiative. The team includes youth, families, health researchers, health funders, and knowledge users from Canada, the United States, and Australia. This proposal is the first research study put forward and endorsed by this Committee. Our study team commits that we will consult and report back to the Research Steering Committee every 3 months on timelines, deliverables, and evaluation metrics, as well as project budget. These frequent reports and consultations will allow for early dissemination of preliminary results. This will also lay groundwork for a process to establish youth-oriented needs and priorities and address them in a timely and feasible manner.
V. Contribution to health care systems.
  This study will provide evidence of Foundry’s impact on community health outcomes for youth with mental health and substance use disorders. In recognition of lack of a widely accepted definitions and measurement of mental health and recovery amongst the youth population, this study will provide an overview of needs from the perspectives of youth. It will highlight strategic, cultural, technical, and structural benefits and challenges associated with patient engagement in health-improvement activities and research. We will conclude this study by offering recommendations for policy and practice to Foundry and other youth health services to support the inclusion of youth across all sites.
